# Picking single-nucleotide polymorphisms in forests

**DOI:** 10.1186/1753-6561-1-s1-s59

**Published:** 2007-12-18

**Authors:** Daniel F Schwarz, Silke Szymczak, Andreas Ziegler, Inke R König

**Affiliations:** 1 Institut für Medizinische Biometrie und Statistik, Universität zu Lübeck, Universitätsklinikum Schleswig-Holstein, Campus Lübeck, Ratzeburger Allee 160, 23538 Lübeck, Germany

## Abstract

With the development of high-throughput single-nucleotide polymorphism (SNP) technologies, the vast number of SNPs in smaller samples poses a challenge to the application of classical statistical procedures. A possible solution is to use a two-stage approach for case-control data in which, in the first stage, a screening test selects a small number of SNPs for further analysis. The second stage then estimates the effects of the selected variables using logistic regression (logReg). Here, we introduce a novel approach in which the selection of SNPs is based on the permutation importance estimated by random forests (RFs). For this, we used the simulated data provided for the Genetic Analysis Workshop 15 without knowledge of the true model.

The data set was randomly split into a first and a second data set. In the first stage, RFs were grown to pre-select the 37 most important variables, and these were reduced to 32 variables by haplotype tagging. In the second stage, we estimated parameters using logReg.

The highest effect estimates were obtained for five simulated loci. We detected smoking, gender, and the parental DR alleles as covariates. After correction for multiple testing, we identified two out of four genes simulated with a direct effect on rheumatoid arthritis risk and all covariates without any false positive.

We showed that a two-staged approach with a screening of SNPs by RFs is suitable to detect candidate SNPs in genome-wide association studies for complex diseases.

## Background

To identify genetic polymorphisms predisposing for a complex disease, genome-wide association studies have become more promising with the advances in technological possibilities. The use of 10 k, 100 k, 300 k or 500 k single-nucleotide polymorphisms (SNP) chips increases the chance of detecting associations between the investigated disease and its causative mutations, while at the same time posing challenges for statistical analyses. Specifically, the availability of a vast number of variables with uncertain dependency structures in comparatively small samples makes the application of classical statistical procedures difficult. A possible approach to dealing with huge numbers of SNPs is to use a two-stage approach. Here, typically, the first stage selects a small number of SNPs for further analysis, whereas the second validates the findings in an independent sample.

The aim of our work is to introduce a novel two-stage approach for large-scale association analysis. Specifically, interesting SNPs are identified in the first stage based on random forests (RFs) [1,2]. The second stage uses an independent sample to estimate the effects of the selected variables using logistic regression (logReg). The application of this approach is demonstrated by analyzing the simulated genome-wide scan for rheumatoid arthritis (RA), which was provided for the Genetic Analysis Workshop (GAW) 15, without knowledge of the true model.

## Methods

### Material

The first replicate of the genome-wide SNP data set and, as phenotype data, RA affection status, gender, lifetime smoking, age at ascertainment, as well as DR alleles from father and mother, were utilized. To mimic a case-control study, we randomly selected one affected sibling per affected pair for the cases and one unaffected sibling per control family for the controls, thus obtaining 1500 cases and 2000 unrelated controls. For the two-stage approach, we randomly split the data into two sets with 750 cases and 1000 controls each.

### First stage of analysis

The first stage of our approach was designed to screen for variables most likely to differentiate between cases and controls. Using the phenotype and genotype information for first of the two data sets, RFs with classification trees (CART) were grown to pre-select the most important variables [3].

The importance of the variables was estimated as the permutation importance in a RF. To this end, the number of correct classifications of the out-of-bag (OOB) cases is calculated in every single tree grown in the forest. Then, the values of the specific variable are randomly permuted in the OOB individuals, and these are then re-classified using these new values. Finally, the number of correct classifications with the permuted values was compared with the number of correct classifications in the original data. The difference between these fractions, averaged over all trees in the RF, gives the permutation importance for the respective variable.

To grow RFs and estimate permutation importance values, we used the software R [4] with the randomForest package by Liaw and Wiener. Because of computational limitations, we were not able to grow one RF containing all variables with estimating importance via the permutation procedure. Instead, 155 RFs were grown based on subsets of 5000 variables, randomly selected without replacement. For every RF, 500 trees were grown with a random selection of 20 variables per node. On average, each variable was contained in a RF 84 times (min = 60, max = 106). The average importance scores across all RFs were used as the global importance of a variable.

Díaz-Uriarte et al. [5] proposed a backward elimination heuristic for RFs to obtain a small set of predictive variables. They calculated importance values for all variables once only. To then select variables, they iteratively fitted RFs; for each iteration, they discarded 20% of the least important variables of the previous variable set and calculated OOB error fractions regarding the remaining variables. They finally selected the set of variables which yielded the lowest OOB error across all iterations. With a similar idea, we applied the following forward-elimination approach:

1. Compute global importance score for every variable as described above.

2. Sort variables according to their score.

3. Grow a RF with the most important variable as single predictor.

4. Compute OOB error for this RF.

5. Add next important variable to the set of predictors and grow a RF.

6. Repeat steps 4 and 5.

On the basis of the resulting OOB prediction error estimates, we chose the smallest set of variables leading to a small prediction error (see below for more information).

To avoid multicollinearity of the variables in the second stage, we applied the haplotype tagging approach by Chapman et al. [6] using the mean estimated coefficient of determination across haplotypes *R*^2 ^≥ 0.5 as criterion for SNP selection.

### Second stage of analysis

The aim of the second step was to obtain valid parameter estimates for the selected variables in a logReg. To reduce the amount of overfitting because of data-dependent variable selection in the first stage, we used an independent data set. Lacking a specific biological hypothesis, an additive genetic effect for each SNP was assumed as recommended [7], and the logReg included all variables that were selected in the first stage. To correct for the multiple testing of the selected variables, nominal *p*-values were adjusted according to the Bonferroni-Holm procedure [8]. Because model parameters were estimated in this stage, stringent external validation of the model is still required. In our study, results are compared with the simulated models.

## Results and discussion

**Figure 1:**
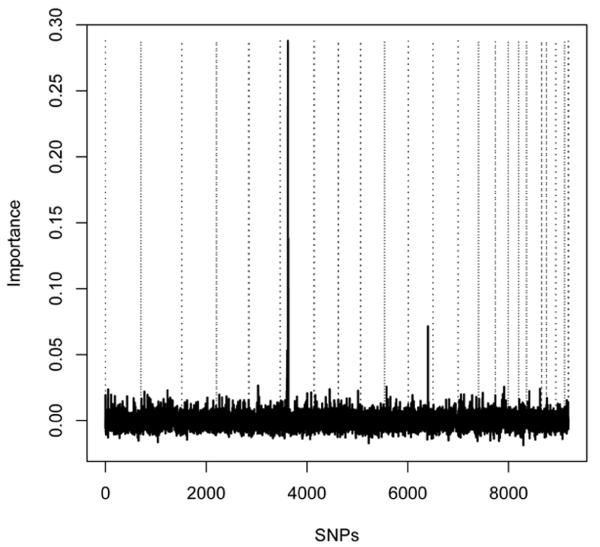
**Importance of SNPs**. Global importance scores for the single SNPs in the genome-wide scan in chromosomal order. Vertical dotted lines show chromosomal boundaries.

Figure 1 shows the global importance scores from the RFs in our first-stage analysis across the genome. It can be seen that highest importance is assigned to SNPs on chromosomes 6 and 11. In addition, high global importance was estimated for phenotypic covariates (not shown). It should be noted that the importance of the covariates might even be underestimated, because the estimated importance in a RF depends on the number of categories of the variable [9]. Specifically, higher importance may be assigned to variables with more categories, and in this case, the covariates were binary in contrast to the SNPs with three categories.

**Figure 2:**
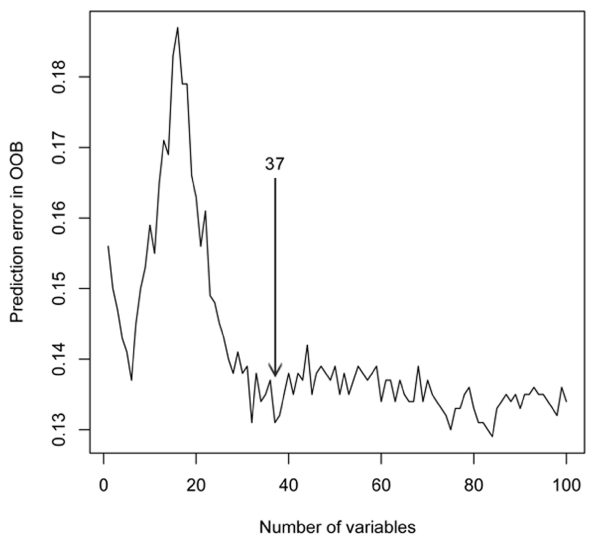
**Prediction error in random forests based on different numbers of variables**. Prediction error of random forests based on different numbers of variables, estimated in the out-of-bag (OOB) samples. Only error estimates of the first 100 sets are displayed. The first local minimum in prediction error is for the set including 37 variables, which was selected for further analyses.

For further analyses, the OOB prediction errors were estimated in RFs with different numbers of variables (Figure 2). It can be seen that with more variables, a strong increase in the estimate is followed by a similarly steep decrease. After this, the error estimate only varies between about 0.13 and 0.14. From this latter region, the point was chosen where the error estimate reaches its first minimum, which is for 37 variables. By haplotype tagging on nine closely neighboring SNPs, this was further reduced to 32 variables for the second stage of analysis.

In the second stage, consideration of all variables in the logReg model (Table 1) shows the largest genetic effects for SNPs on chromosomes 6, 11, and 18. Of these, the regions on chromosomes 6 and 18 correspond to the loci C/DR, D, and E, which were the only ones simulated to have a direct effect on RA risk. One of them, which coincides with loci C/DR, remains significant after adjustment for multiple testing. In addition, the region on chromosome 11 identifies locus F, which has an indirect effect on RA risk via IgM level. Finally, we identified the paternal and maternal DR alleles to increase the chance for affection, and the analyses provide evidence for female gender and lifetime smoking contributing to a higher chance of disease. There were no false-positive findings.

**Table 1:** Effect estimates of the selected variables

	logReg^a^				
		
Variable	Coef	SE	nominal *p*	adjusted *p*	Simulated effects
DR allele from mother	1.476	0.114	<10^-15^	<10^-13^	
DR allele from father	1.480	0.115	<10^-15^	<10^-13^	
Chr 11 bp110, 204, 257	0.822	0.123	2.40 × 10^-11^	7.20 × 10^-10^	Locus F
Lifetime smoking	0.976	0.168	7.06 × 10^-9^	2.05 × 10^-7^	Smoking
Gender	0.804	0.169	2.14 × 10^-6^	5.99 × 10^-5^	Gender
Chr 6 bp 32, 521, 277	-0.861	0.201	1.87 × 10^-5^	0.0005	DR/Locus C
Chr 18 bp 66, 048, 927	0.333	0.133	0.0121	0.3146	Locus E
Chr 6 bp 36, 582, 440	0.634	0.255	0.0131	0.3275	Locus D
Chr 6 bp 28, 758, 332	0.248	0.125	0.0477	1.0000	
Chr 1 bp 26, 043, 914	0.185	0.143	0.1942	1.0000	
Chr 2 bp 34, 451, 973	0.154	0.126	0.2228	1.0000	
Chr 6 bp 30, 266, 243	-0.184	0.165	0.2647	1.0000	
Chr 7 bp 97, 632, 608	0.116	0.125	0.3522	1.0000	
Chr 6 bp 26, 075, 047	-0.126	0.138	0.3613	1.0000	
Chr 18 bp 10, 152, 707	0.098	0.115	0.3907	1.0000	
Chr 8 bp 127, 252, 736	-0.101	0.121	0.4050	1.0000	
Chr 13 bp 45, 600, 085	-0.212	0.258	0.4094	1.0000	
Chr 13 bp 31, 890, 164	0.090	0.116	0.4356	1.0000	
Chr 11 bp 22, 794, 066	0.118	0.155	0.4475	1.0000	
Chr 5 bp 57, 110, 585	-0.251	0.337	0.4559	1.0000	
Chr 6 bp 32, 772, 203	0.094	0.133	0.4769	1.0000	
Chr 4 bp 15, 714, 556	0.066	0.112	0.5547	1.0000	
Chr 6 bp 133, 756, 692	0.072	0.133	0.5885	1.0000	
Chr 14 bp 37, 328, 424	0.073	0.142	0.6051	1.0000	
Chr 1 bp 48, 687, 156	-0.192	0.378	0.6115	1.0000	
Chr 15 bp 77, 852, 281	0.097	0.195	0.6170	1.0000	
Chr 10 bp 10, 764, 908	0.050	0.133	0.7034	1.0000	
Chr 15 bp 66, 671, 014	0.049	0.222	0.8235	1.0000	
Chr 2 bp 17, 889, 207	0.059	0.269	0.8261	1.0000	
Chr 6 bp 155, 580, 230	-0.020	0.131	0.8757	1.0000	
Chr 7 bp 8, 524, 374	-0.009	0.116	0.9332	1.0000	
Chr 2 bp 157, 502, 490	0.018	0.570	0.9744	1.0000	
Intercept	-11.464	1.594			

^a^logReg, logistic regression; Coef, estimated regression coefficient; SE, standard error, nominal *p*, two-sided nominal *p*-value; adjusted *p*, two-sided *p*-value adjusted according to the Bonferroni-Holm procedure; chr, chromosome; bp, base pair.

To summarize, our results show that RFs can be applied as a pre-screening tool in genome-wide association studies. Our two-staged approach with a selection of SNPs by RFs is suitable to detect promising candidate SNPs in large-scale association studies for complex diseases.

## Competing interests

The author(s) declare that they have no competing interests.

## References

[B1] Lunetta K, Hayward L, Segal J, Eerdewegh P (2004). Screening large-scale association study data: exploiting interactions using random forests. BMC Genet.

[B2] Heidema A, Boer J, Nagelkerke N, Mariman E, van der AD, Feskens E (2006). The challenge for genetic epidemiologists: how to analyze large numbers of SNPs in relation to complex diseases. BMC Genet.

[B3] Breiman L (2001). Random forests. Mach Learn.

[B4] The R Project for Statistical Computing. http://www.r-project.org/.

[B5] Diaz-Uriarte R, Alvarez de Andres S (2006). Gene selection and classification of microarray data using random forest. BMC Bioinformatics.

[B6] Chapman J, Cooper J, Todd J, Clayton D (2003). Detecting disease associations due to linkage disequilibrium using haplotype tags: a class of tests and the determinants of statistical power. Hum Hered.

[B7] Ziegler A, König I (2006). A Statistical Approach to Genetic Epidemiology.

[B8] Westfall PH, Young SS (1993). Resampling-Based Multiple Testing.

[B9] Strobl K, Boulesteix A-L, Zeileis A, Hothorn T (2007). Bias in random forest variable importance measures: illustrations, sources and a solution. BMC Bioinformatics.

